# Cancer risks of dermatomyositis and polymyositis: a nationwide cohort study in Taiwan

**DOI:** 10.1186/ar2987

**Published:** 2010-04-16

**Authors:** Yi-Ju Chen, Chun-Ying Wu, Yu-Lin Huang, Chang-Bi Wang, Jui-Lung Shen, Yun-Ting Chang

**Affiliations:** 1 Department of Dermatology, National Yang-Ming University, No. 155, Sec. 2, Linong Street, Taipei 112, Taiwan; 2 Department of Dermatology, Taichung Veterans General Hospital, No. 160, Sec. 3, Chung-Kang Road, Taichung 40705, Taiwan; 3 Department of Internal Medicine, National Yang-Ming University, No. 155, Sec. 2, Linong Street, Taipei 112, Taiwan; 4 Department of Gastroenterology, Taichung Veterans General Hospital, No. 160, Sec. 3, Chung-Kang Road, Taichung 40705, Taiwan; 5 Institute of Public Health, China Medical University, No. 91 Hsueh-Shih Road, Taichung 40402, Taiwan; 6 Department of Dermatology, Taipei Veterans General Hospital, No. 201, Sec. 2, Shih-Pai Road, Taipei 112, Taiwan

## Abstract

**Introduction:**

The association of idiopathic inflammatory myositis (IIM) and malignancies has been reported, but rarely in Asian countries. Our aim was to investigate the risk of cancer among IIM patients without a prior history of malignancies, in Taiwan.

**Methods:**

We conducted a nationwide cohort study of 1,012 patients with dermatomyositis (DM) and 643 patients with polymyositis (PM), but without prior history of malignancies, utilizing the National Health Insurance Database from 1997 to 2007. Standardized incidence ratios (SIRs) of cancers were analyzed.

**Results:**

A total of 95 cancers (9.4%) in DM and 33 cancers (4.4%) in PM were identified. Overall cancer risk was significantly elevated in DM patients (SIR = 5.11, 95% confidence interval [CI] = 5.01 to 5.22) and PM patients (SIR = 2.15, 95% CI = 2.08 to 2.22). Most cancers were detected in the first year of observation. The risk of cancer decreased with observation time, yet remained elevated compared with the general population in both study groups after 5 years of follow-up. DM was associated with sustained elevated risk of cancers in every age group, whereas the risk of cancer in PM was highest in younger patients and decreased with age. DM patients were at the greatest risk of cancers of the nasopharynx, lungs and hematopoietic malignancies.

**Conclusions:**

Patients with IIM are at increased risk for cancer and should receive age-appropriate and gender-appropriate malignancy evaluations, with additional assessment for nasopharyngeal, lung and hematologic malignancy following diagnosis, and with continued vigilance for development of cancers in follow-up.

## Introduction

The association of malignancies with dermatomyositis (DM) or polymyositis (PM) has been studied extensively both in Caucasian and Asian populations. Population-based cohort studies in Denmark, Sweden and Finland [1,2] have demonstrated that DM, and to a lesser extent PM, carries elevated risk of comorbid cancers, in which cancers of the ovary, lung, pancreas, stomach and urinary bladder, as well as hematologic cancers including non-Hodgkin's lymphoma and Hodgkin's lymphoma, are the most relevant [1]. Most comorbid cancers are detected within the first year of diagnosis of idiopathic inflammatory myositis (IIM) [1,2]. A thorough physical and laboratory work-up for early detection of these cancers is therefore strongly recommended after diagnosis of IIM.

The epidemiologic data on IIM-associated cancer types, however, are scarce and varied in Asian populations. Breast cancer, stomach cancer and nasopharyngeal cancer (NPC) have been reported to be more commonly associated with DM in Korea [3]. Studies in Singapore, Hong Kong, southeastern China and Taiwan have revealed that NPC is the most common cancer associated with DM [4-7]. Our recent population-based, case-control study demonstrated that DM and PM patients are associated with a sixfold to 10-fold increased chance of cancer compared with control subjects. About two-thirds of comorbid cancers occur after diagnosis of IIM [8].

The present study aimed to investigate the risk of specific cancer types among a DM/PM cohort by IIM subtype, age at diagnosis, and follow-up time, utilizing analyses of standardized incidence ratios (SIRs).

## Materials and methods

### Data sources

The present study was based on data from the National Health Insurance Research Database released by the National Health Research Institute. Taiwan began its National Health Insurance program in 1995 to finance healthcare for all of its residents. There are currently >25 million enrollees in the program, representing approximately 99% of Taiwan's entire population. The database comprises comprehensive information on insured subjects, such as demographic data, dates of clinical visits, diagnostic codes, details of prescriptions, and expenditure amounts. This database has been the source of many epidemiological studies published in peer-reviewed journals [8-13].

International Classifications of Disease-9 (ICD-9) codes were used to define diseases during the study period. Personal information, including family history, lifestyle and habits such as smoking and alcohol use, was not available from the National Health Insurance Research Database.

### Study subjects

All enrollees were obtained from the Registry of Catastrophic Illness Database, a subpart of the National Health Insurance Research Database. The insured who suffer from major diseases can apply for a catastrophic illness certificate, which grants exemption from co-payment. DM, PM and cancer are statutorily included in the catastrophic illness category. Both outpatient and inpatient claims of beneficiaries with a catastrophic illness registry are collected in the catastrophic illness profile and distributed as a package.

Application of catastrophic illness certificate for DM and PM requires a thorough clinical and laboratory survey that fulfills the diagnostic criteria proposed by Bohan and Peter [14,15]. The diagnostic criteria for DM and PM include: symptoms of proximal muscle weakness; abnormal results on electromyography; abnormal results on muscular biopsy; elevated serum levels of muscle enzyme (creatine kinase), lactate dehydrogenase, aspartate transaminase and alanine transaminase; and typical skin rash compatible with DM. Only those with diagnosis of definite DM or definite PM are issued a catastrophic illness certificate.

The enrollees with DM (ICD-9 code 710.3) and with PM (ICD-9 code 710.4) were followed up between 1 January 1997 and 31 December 2007. Amyopathic DM and inclusion body myositis were not identified due to a lack of specific diagnostic codes.

As the dataset used in this study consists of de-identified secondary data released to the public for research purposes, the study was exempt from full review by the Institutional Review Board.

### Identification of cancer cases

To apply for a cancer catastrophic illness certificate, cytological or pathological reports or evidence such as additional laboratory and image studies supporting the diagnosis of cancer - including tumor marker surveys, X-ray scan, bone scan, computed tomography scan or magnetic resonance imaging scan - should be provided. We excluded those patients with *in situ *malignancies because *in situ *malignant diseases do not qualify for a catastrophic illness certificate. The diagnostic codes of malignancies were defined as those from code 140 to code 208.91 in the ICD-9 Clinical Modification format.

### Cancer risk analysis

All enrolled study subjects were followed up until a first-time diagnosis of cancer (except malignancy *in situ *or metastatic cancers), death, the end of follow-up in the medical records, the end of the observation period, or the end of 2007. SIRs of cancers were analyzed. Stratified analyses according to age at diagnosis, gender, and years of follow-up were conducted.

### Statistical analysis

The demographic data of the study population were first analyzed. Follow-up for each patient with myositis began at the date of diagnosis and ended at the date of censorship - that is, the date of diagnosis of cancer, death or the end of follow-up period - and was measured in numbers of years. We examined the associations among DM, PM and specific cancer types with SIRs. The SIR was calculated as follows: the number of cancer cases that arose among DM or PM patients divided by the expected number of cancer cases according to national age-specific, gender-specific, and period-specific cancer rates. Yearly reports of cancer rates were obtained from the Taiwan National Cancer Registry.

To assess the age effect on the relative risk of malignancies, we analyzed the relative risks among those aged 0 to 19 years, 20 to 39 years, 40 to 59 years, 60 to 79 years, and >80 years at myositis diagnosis. A further analysis was performed to evaluate whether the association of malignancies varied according to the time after myositis diagnosis. We divided the follow-up time into four periods: ≤ 1 year, 1 to 2 years, 2 to 5 years and ≥ 5 years.

The SAS statistical package (SAS System for Windows, version 9.1; SAS Institute, Cary, NC, USA) and SPSS statistics (SPSS Statistics for Windows, version 15.0; SPSS Inc., Chicago, IL, USA) were used to perform the statistical analysis of the data in the present study.

## Results

We identified a total of 1,012 patients with DM and 643 patients with PM, all without previous malignancies. The mean ± standard deviation ages at diagnosis of DM and PM were 41.79 ± 18.96 years and 48.38 ± 16.63 years, respectively. Among DM patients, 315 (31.13%) were male and 697 (68.87%) were female. There were 199 (30.94%) male and 444 (69.06%) female patients in the PM group (Table 1). The mean ± standard deviation follow-up times for DM and PM were 5.09 ± 3.72 years and 5.05 ± 3.42 years, respectively.

**Table 1:** Age and gender distributions of the dermatomyositis and polymyositis study groups

Age	Dermatomyositis group (n = 1,012)			Polymyositis group (n = 643)		
		
	Number of cases	Male	Female	Number of cases	Male	Female
<20 years	162	50	112	31	14	17
20 to 39 years	265	76	189	161	45	116
40 to 59 years	414	120	294	274	76	198
60 to 79 years	164	66	98	169	61	108
≥ 80 years	7	3	4	8	3	5
All	1,012	315	697	643	199	444

A total of 95 cancers (9.4%) in DM patients and 33 cancers (4.4%) in PM patients were identified after diagnosis of myositis. The overall cancer risk was significantly elevated among patients with DM (SIR = 5.11, 95% confidence interval [CI] = 5.01 to 5.22), and to a lesser extent among patients with PM (SIR 2.15, 95% CI = 2.08 to 2.22) (Table 2). The SIRs for men and women with DM were 5.44 (95% CI = 5.28 to 5.62) and 5.10 (95% CI = 4.96 to 5.23), respectively. Among PM patients, women had a slightly higher risk than men for the development of malignancies (SIR = 2.36 for women, SIR = 1.84 for men).

**Table 2:** Cancer incidence among dermatomyositis and polymyositis patients by age, gender and years of follow-up

	Dermatomyositis group (n = 1,012)			Polymyositis group (n = 643)		
		
	Observed	Expected	SIR (95% CI)	Observed	Expected	SIR (95% CI)
Total	95	18.58	5.11 (5.01 to 5.22)	33	15.36	2.15 (2.08 to 2.22)
Age						
<20 years	1	0.13	7.86 (6.26 to 9.36)	0	0.02	-
20 to 39 years	8	1.11	7.20 (6.72 to 7.72)	7	0.65	10.69 (9.99 to 11.60)
40 to 59 years	55	6.67	8.25 (8.03 to 8.47)	13	4.98	2.61 (2.47 to 2.76)
60 to 79 years	29	5.40	5.37 (5.18 to 5.57)	13	6.80	1.91 (1.81 to 2.02)
≥ 80 years	2	0.24	8.47 (7.22 to 9.57)	0	0.52	-
Gender						
Male	39	7.16	5.44 (5.28 to 5.62)	12	6.51	1.84 (1.74 to 1.95)
Female	56	10.99	5.10 (4.96 to 5.23)	21	8.89	2.36 (2.26 to 2.47)
Follow-up						
<1 years	64	3.00	21.30 (20.81 to 21.86)	18	2.67	6.75 (6.43 to 7.06)
1 to 2 years	12	2.36	5.08 (4.80 to 5.38)	3	2.30	1.31 (1.16 to 1.46)
2 to 5 years	13	5.16	2.52 (2.38 to 2.66)	5	4.93	1.01 (0.93 to 1.11)
>5 years	6	4.39	1.37 (1.26 to 1.48)	7	3.51	1.99 (1.85 to 2.15)

Expected cancer cases were calculated by age-specific and gender-specific estimates of the general population in Taiwan 2006. CI, confidence interval; SIR, standardized incidence ratio.

With regards to age, a bimodal-pattern distribution of malignancy risk was observed among DM patients. DM patients carried a sustained elevated risk of cancer in every age group, especially in those younger than 60 years and those older than 80 years (Table 2). For PM, younger patients - especially those in their 20s - carried the highest risk of malignancies (SIR = 10.69, 95% CI = 9.99 to 11.60). The malignancy risk was reduced in patients with older age at diagnosis (SIR = 2.61 in those 40 to 59 years old, SIR = 1.91 in those ≥ 60 years old).

Comorbid malignant diseases were mostly detected following the first year of diagnosis, both in DM patients and in PM patients (SIR = 21.30 and SIR = 6.70, respectively). The risk of cancer decreased with follow-up time. For example, the SIRs of cancer in DM during the 1 to 2 years and the 2 to 5 years of observation were 5.08 (95% CI = 4.80 to 5.38) and 2.52 (95% CI = 2.38 to 2.66), respectively. The risk of cancer in PM was reduced to the expected incidence ratios of the general population after 2 years of follow-up (Table 2). The risk remained elevated both in DM and PM patients after 5 years of observation, however, with SIR = 1.37 (95% CI = 1.26 to 1.48) and SIR = 1.99 (95% CI = 1.85 to 2.15), respectively.

The highest risks after diagnosis of DM were for NPC, lung/mediastinum cancers, bone/joint cancers and lymphomas/leukemias. An elevated risk for many other cancers was also observed in DM patients (Table 3). For PM patients, although there were limited cancer cases, the greatest risks were for cancers of the bone and joint, the brain, NPC and melanoma. Elevated risks of leukemia/lymphomas and cancers of the uterus, oropharynx/larynx, urinary bladder, liver, colorectum and pancreas were also observed in PM patients.

**Table 3:** Incidence for specific cancer types after diagnosis of dermatomyositis and polymyositis

Cancer type	Dermatomyositis group (n = 1,012)			Polymyositis group (n = 643)		
		
	Observed^a^	Expected^b^	SIR (95% CI)	Observed^a^	Expected^b^	SIR (95% CI)
Nasopharynx	30	0.21	139.94 (137.79 to 148.06)	2	0.16	12.71 (10.83 to 14.36)
Lung^c^	22	1.07	20.58 (19.71 to 21.44)	5	0.93	5.38 (4.92 to 5.87)
Breast	9	2.55	3.53 (3.30 to 3.77)	3	1.80	1.67 (1.48 to 1.87)
Uterus	0	-	-	1	0.14	7.13 (5.81 to 8.69)
Uterine cervix	3	0.91	3.28 (2.93 to 3.68)	1	0.70	1.44 (1.16 to 1.74)
Ovary^c^	2	0.38	5.33 (4.56 to 6.05)	0	-	-
Lymphoma/leukemia	4	0.16	24.70 (22.61 to 27.57)	1	0.13	7.78 (6.26 to 9.36)
Oropharynx and larynx	1	0.13	7.46 (6.26 to 9.36)	0	-	-
Esophagus	1	0.33	3.06 (2.47 to 3.69)	0	-	-
Liver/gall bladder	5	1.38	3.62 (3.31 to 3.96)	4	1.20	3.34 (3.01 to 3.68)
Colorectum	5	1.21	4.12 (3.78 to 4.51)	5	1.06	4.70 (4.31 to 5.15)
Stomach	1	0.97	1.03 (0.84 to 1.25)	0	-	-
Pancreas	1	0.33	3.04 (2.47 to 3.69)	1	0.29	3.44 (2.81 to 4.19)
Kidney	3	0.51	5.93 (5.24 to 6.59)	1	0.45	2.22 (1.81 to 2.70)
Urinary bladder	2	0.49	4.05 (3.54 to 4.69)	2	0.44	4.59 (3.94 to 5.22)
Melanoma	1	0.23	4.33 (3.54 to 5.29)-	2	0.20	9.76 (8.66 to 11.49)
Bone/joint	1	0.07	14.87 (11.62 to 17.38)	2	0.05	39.64 (34.65 to 45.94)
Brain	0	-	-	2	0.11	18.21 (15.75 to 20.88)
Thyroid	2	0.45	4.49 (3.85 to 5.10)	1	0.32	3.12 (2.54 to 3.80)
Metastatic cancers^c^	2	0.56	3.57 (3.09 to 4.10)	0	-	-

^a^Observed cancer cases. ^b^Expected cancer cases based on estimates of the general population in Taiwan, after age and gender adjustment. ^c^Lung, including lung and mediastinum; ovary, including ovary and fallopian tube; metastatic cancers, including cancers of ill-defined or unknown origin. CI, confidence interval; SIR, standardized incidence ratio.

## Discussion

A strong association between malignancy and myositis, especially in DM patients, was observed in our study. Most cancer cases were detected within the first year after diagnosis. This observation was consistent with the majority of reports in the literature, suggesting a paraneoplastic nature for IIM. Aggressive surveillance for cancer during that period may have resulted in a detection bias. When cancer cases observed in the first 2 years were excluded, a sustained elevated risk existed among DM patients after 5 years of follow-up (SIR = 1.37) (Table 2) - indicating a true link between these two diseases. The risk of malignancy in PM was reduced to the expected incidence ratio of the general population at 2 years of observation. Although there appeared to be an increased risk of cancer after 5 years of follow-up, the risk could not be adequately assessed because of the small number of cases, resulting in unstable estimates of risk. The lifelong use of immunosuppressive drugs in both diseases may also contribute to an elevated risk of cancer after years of observation. The relationship between cancer and PM needs further exploration. Overall, we suggest that physicians continue to carry out cancer work-ups in myositis patients for at least 5 years in IIM patients.

Only a few reports have focused on the age effect of cancer risk among DM and PM patients. Hill and colleagues indicated increased risks of cancer among DM patients younger than 44 years (SIR = 2.2) and older than 45 years (SIR = 3.1) [1]. There was no such trend, however, in PM patients. Another report from Stockton and colleagues indicated that cancer risk decreases with age in DM patients, but not in PM patients [16]. Those authors did not, however, further discuss the age effect.

In the present article, we demonstrated that the relative risk of cancer in DM remains high in every age group, whereas the risk of cancer in PM is highest in younger patients and decreases with age. Since malignant diseases increase with age among the general population (data not presented), it is reasonable that the higher prevalence of cancer cases among aged patients makes the difference in incidence nonsignificant between older PM patients and older people from the general population. Moreover, the sustained elevated cancer risk in DM patients highlighted the true link between DM and malignancies despite the age effect.

A strong link between DM and NPC was highlighted in the present study, with the SIR reaching as high as 140. A pathogenetic correlation between cancers of the pharynx, especially NPC, and DM has been proposed. NPC is endemic to southeastern Asia and is related to the chronic active infection of Epstein-Barr virus [17-19]. Three cases of chronic active Epstein-Barr virus infection-induced generalized myositis have been reported [19]. Furthermore, Epstein-Barr virus has been detected in lung specimens from patients with rapidly progressive interstitial pneumonitis in PM/DM [20]. The immune response to Epstein-Barr virus contributes to the coexistence of DM/PM and NPC.

Other comorbid cancers commonly associated with DM are cancers of the lungs/mediastinum, bone/joints and kidney, as well as lymphoma and leukemia, with the SIRs ranging from 5 to 24. In contrast to the most commonly associated cancers, such as those of the lungs and ovaries, as well as lymphoma and leukemia, cases of myositis associated with renal or adrenal carcinomas have rarely been reported [21-24]. Symptoms and laboratory abnormalities of DM or PM have been reported to be alleviated after surgical intervention for renal cancers [21,22]. In addition, there are rare occurrences of chondrosarcoma [25], osteosarcoma [26] and thyroid cancers [27,28] in DM; yet the relationship between these diseases and DM remains unclear.

Several population-based studies have indicated that ovarian cancer is the most commonly associated malignancy in female DM patients, with SIRs ranging from 8.2 to 32 [1,2,29,30], whereas the correlation between ovarian cancer and PM is low. Ovarian carcinoma, however, has not been addressed in studies from Asian populations [4,6,31]. The risk of ovarian cancer was elevated in our DM patients, although with a lower SIR (5.33) than in western populations. Only two cases of ovarian cancer were observed in our DM patients, and no cases were observed in our PM patients. Mordel and colleagues reviewed published case reports of ovarian cancer and DM, and revealed that those with DM had a higher rate of advanced ovarian adenocarcinoma (96%) than the general female population with ovarian carcinoma (60 to 70%) [32].

Although there were limited numbers, diverse comorbid cancer types were associated with PM. The most significantly associated cancers included cancers of the bone/joints, nasopharynx and brain, and melanoma. The correlation between these cancers and myositis, however, remains unclear. Malignant melanoma-complicated DM/PM has been frequently reported [33-37] and the development of myositis in patients with melanoma may serve as a poor prognostic marker [33,35]. Very few reports have mentioned the correlation of these cancers in PM. Actually, the true association of these cancers in PM is doubtful and the risk of these cancers may be overestimated in the present study due to the very small number of cases.

An underlying mechanism of the association between IIM and malignancies remains unclear. A model of crossover immunity for the development of cancer-associated myositis has been proposed recently [38-40]. Common antigenic myositis-specific autoantigens were expressed in both tumor cells and undifferentiated myoblasts. Myositis-specific autoantigen expression in a nascent tumor leads to the generation of both specific T cells and B cells against those antigens, and then to successful anti-tumor immunity. In a subset of patients, subsequent muscle damage from a variety of potential causes (such as viral infection, trauma, toxin exposure) may lead to muscle damage and regeneration, and may reactivate immune responses previously generated in the initial anti-tumor response. The crossover immunity between tumor cells and myofibroblasts may result in the parallel clinical course of both diseases [41-44].

Until now, no population-based studies have provided evidence for the causal relationship between IIM and malignancies. A lower risk of cancer has been reported in patients who previously received cytotoxic treatment for inflammatory myopathies [29,45]. In the present article, we demonstrated that the cancer risk in myositis patients decreases with time - which favors the role of the inflammatory nature, rather than lifelong immunosuppressive treatment, in the development of malignancies.

A limitation of the registry-based database is the possible misclassification of IIM. Due to the lack of specific diagnostic codes, amyopathic DM and inclusion body myositis - which have been reported to be associated with malignancies - were not identified in our study. The risk of cancer in IIM might therefore be underestimated. An individually tailored screening for malignancy in IIM patients has been recommended according to age, sex, ethnicity and subset of IIM. In brief, we strongly suggest conducting a thorough physical and laboratory check-up for detecting malignancies for at least 5 years following the diagnosis of IIM, especially in DM patients. In addition to the lungs and ovaries and lymphoma/leukemia, we recommend that special attention be paid to the risk of NPC and cancers of the renal system in DM and PM patients. A nasopharyngeal endoscopy examination should be routinely performed in all IIM patients. A thorough physical examination including palpation of thyroid glands, breast and lymph nodes will assist in the early detection of occult cancers. Mammography, breast ultrasonography and the Papanicolaou test are essential in female IIM patients. A more detailed history-taking and physical examinations, including symptoms of headache, neurologic signs, soreness of bones and joints, and skin examination, should be mandatory in PM patients.

## Conclusions

Patients with DM and PM carry an elevated risk of various cancers in Taiwan. We suggest thorough physical and laboratory tests for malignancies, especially those of the nasopharynx, for at least 5 years of follow-up.

## Abbreviations

CI: confidence interval; DM: dermatomyositis; ICD-9: International Classifications of Disease 9; IIM: idiopathic inflammatory myositis; NPC: nasopharyngeal carcinoma; PM: polymyositis; SIR: standardized incidence ratio.

## Competing interests

The authors declare that they have no competing interests.

## Authors' contributions

YJ-C conceived the study, participated in its design, performed the statistical analysis and interpretation of the data, and drafted the manuscript. CY-W participated in the design of the study, acquisition of the funding, interpretation of the data, and drafting of the manuscript. Y-LH participated in the design of the study and helped to draft the manuscript. CB-W performed the statistical analysis and participated in the design of the study. J-LS participated in the design of the study. YT-C conceived the study, participated in its design, and supervised the drafting of the manuscript.
